# Constrained Unfolding of a Helical Peptide: Implicit versus Explicit Solvents

**DOI:** 10.1371/journal.pone.0127034

**Published:** 2015-05-13

**Authors:** Hailey R. Bureau, Dale R. Merz, Eli Hershkovits, Stephen Quirk, Rigoberto Hernandez

**Affiliations:** 1 Center for Computational and Molecular Science and Technology, School of Chemistry and Biochemistry, Georgia Institute of Technology, Atlanta, Georgia 30332-0400, United States of America; 2 Kimberly-Clark Corporation, Atlanta, GA 30076-2199, United States of America; Instituto de Tecnologica Química e Biológica, UNL, PORTUGAL

## Abstract

Steered Molecular Dynamics (SMD) has been seen to provide the potential of mean force (PMF) along a peptide unfolding pathway effectively but at significant computational cost, particularly in all-atom solvents. Adaptive steered molecular dynamics (ASMD) has been seen to provide a significant computational advantage by limiting the spread of the trajectories in a staged approach. The contraction of the trajectories at the end of each stage can be performed by taking a structure whose nonequilibrium work is closest to the Jarzynski average (in naive ASMD) or by relaxing the trajectories under a no-work condition (in full-relaxation ASMD—namely, FR-ASMD). Both approaches have been used to determine the energetics and hydrogen-bonding structure along the pathway for unfolding of a benchmark peptide initially constrained as an α-helix in a water environment. The energetics are quite different to those in vacuum, but are found to be similar between implicit and explicit solvents. Surprisingly, the hydrogen-bonding pathways are also similar in the implicit and explicit solvents despite the fact that the solvent contact plays an important role in opening the helix.

## Introduction

The numerical determination of the free energy for unfolding large peptides remains a major computational challenge. The requisite integration time is long because the ends of the protein must be pulled to a very long distance at relatively slow pulling speeds. The computational requirements are further exacerbated by the inclusion of an explicit solvent box that has to be large enough to contain the unfolded and folded protein structures with room to spare for solvent shells. As the end-to-end length of the protein grows, the number of required water molecules also grows, leading to ever larger computer memory requirements. These costs can be mitigated through the use of implicit solvent models, but there has been much debate over how well they can mimic explicit solvent effects. [1]

**Fig 1:**
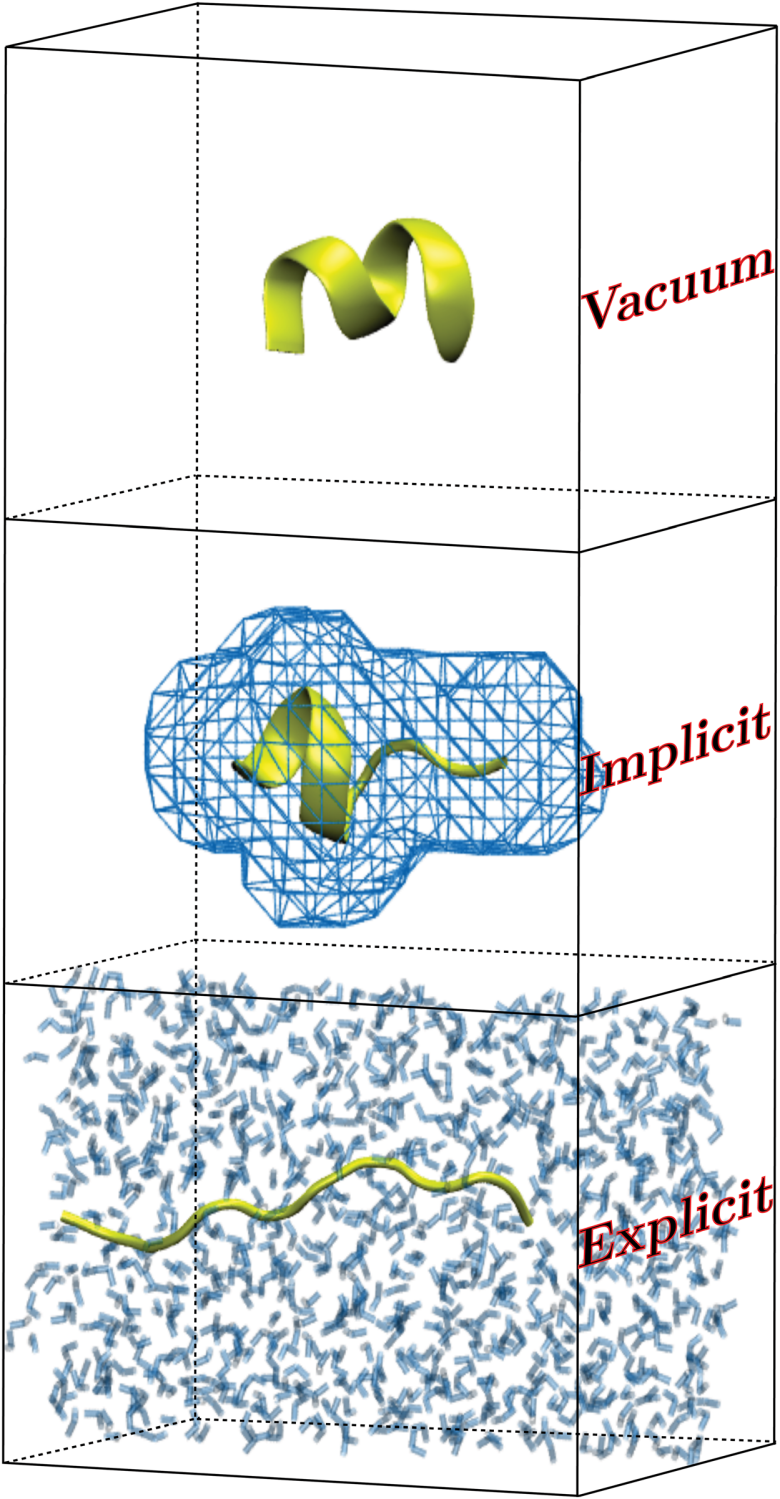
An illustration of the three solvent regimes that are considered for the solvation of ALA_10_ in this work: vacuum (top), implicit solvent (center), and explicit TIP3P water solvent (bottom). In each frame, the ALA_10_ peptide is shown in a different configuration along the pulling coordinate.

Skinner and coworkers, [2, 3] for example, found that the environment plays a critical role in the structure formation of the putative helical segment in Rat and Human Amylin, but the question remains as to whether the solvent must be specified at all-atom resolution in order to obtain such effects in every case. The early work of Paci and Karplus, [4] assumed that an implicit solvent suffices in studying the structure and energetics for pulling titin. However, subsequent work on small alkanes [5] and ALA_10_ [6] suggests that the water model representing the solvent needs to be considered at least at a coarse-grained level so as to obtain the correct energetics and structure along the pathway. In our recent work, [7] we found that the energetics and the hydrogen-bonding pathway along the stretching of ALA_10_—whose structures are illustrated in Fig 1,—was quite different between vacuum and explicit solvents. However, Gumbart and coworkers [8] recently found a PMF for ALA_10_ in explicit water that differed from our previous result. As such, the determination of the correct pathway presents a significant test of the ASMD approach as well as the use of an implicit solvent model. Surprisingly, in the present work, we found that the overall pathway, in which internal hydrogen-bonds are broken and supplanted by contact to the solvent, is mostly recovered by the implicit solvent.

The use of implicit solvent models has gained popularity because they provide the possibility for reducing a large number of degrees of freedom (as it effectively coarse-grains the solvent away) and thereby reduce the computational cost significantly. They have been seen to yield relatively accurate predictions of protein native structure [9, 10] and even folding pathways of short peptides. [11–13] Luchko et al [14] provides a recent comparison of the relative accuracy in the energetics of several implicit solvent models. Earlier numerical experiments of a *β*-hairpin folding by Zhou and coworkers [15] found that the folding structure depended heavily on the choice of the GBSA force field; only the AMBER96/GBSA force field gave the known native structure. They also found that the implicit solvent models tend to elongate helices and reduce the stability of the *β*-hairpins relative to the native structure. Nilsson and coworkers [16] performed MD studies at four different temperatures in ten different implicit solvent models implemented in CHARMM. They found that a variant of the GBIS model—namely Generalized Born using molecular volume GBMV II—was the best choice for captutring the solvation energetics in unfolding and folding simulations. There now exists ample evidence [9, 17] that implicit models can be used to obtain accurate near-equilibrium structures as long as a surface-dependent term is included in the representation. The question explored in the present work is whether this is also sufficient to obtain the correct PMF and structural pathway through SMD-like calculations.

Steered Molecular dynamics (SMD) is a useful tool for evaluating the free energy of transitions between different states of a molecule. [18, 19] SMD is based on the numerical calculation of the average (via the Jarzynski equality [20–22]) over the nonequilibrium distribution of exponentials of the negative work performed while the molecule is pulled along a specific path. The Jarzynski equality can, in principle, also be used in conjunction with multiple single molecule pulling experiments to construct the free energy profile. [23, 24] SMD has been used to determine the free energy profile for a number of different types of systems, for example, to find the unfolding free energy of a protein [18] or to understand the dissociation of a salt compound in a solvent. [25] One of the main drawbacks of SMD is that the logarithm of an average of exponentials (which we call the “exponential average”) of the work is much lower than the average of the work. Hence, the trajectories with the greatest contribution to the Jarzynski average are those with the lowest work and these are necessarily rare events.

Finding the rare events contributing to the exponential average requires substantial sampling over a very large space. Attempts to reduce the number of trajectories for a correct estimation of the free energy include: (i) The use of the backward as well as the forward trajectories leading to improved convergence in the averaging of the work. [26, 27] This can be further enhanced through the use of the maximum likelihood estimator (MLE). [28, 29] (ii) The use of multistage methods so as to decrease divergence of the work functions of trajectories at increasing time. These include Kofke’s multistage approach, [30] multistep trajectory combination (MSTC), [31, 32] our own Adaptive Steered Molecular Dynamics (ASMD) method, [7, 33, 34] and several others. [35–37] These methods share a similar strategy in that they are all based on segmenting the pulling path into several smaller stages and independently calculating the Jarzynski average along these stages. The advantage of this segmentation lies in the fact that a smaller stage leads to a smaller span of trajectories which can be more readily fully sampled despite the possibility of rare low-work trajectories.

Naive ASMD and full-relaxation ASMD (FR-ASMD) follow the second of these strategies. In both of these methods, the major advantage involves the contraction of trajectories at the end of a stage to structures closer to those representative of the equilibrium distribution of the peptide constrained to the particular extension. In naive ASMD, a single structure is chosen from those in the numerically determined nonequilibrium ensemble according to which had the associated work closest to the Jarzynski average. [33, 34] In FR-ASMD, all of the trajectories are relaxed for some additional integration under the constrained extension. [31, 34] As this additional constrained nonequilibrium path requires no work on the system, the Jarzynski average is unaffected through the relaxation stages of FR-ASMD. For example, in previous work, we found that ASMD could be used to obtain the unfolding free energy for ALA_10_ in vacuum [34] and explicit water solvent [7] achieving apparent convergence with relatively few trajectories.

In this work, we compare the PMFs of ALA_10_ in vacuum, implicit, and explicit solvent using SMD, ASMD, and FR-ASMD. Although this is not the first time that the PMF has been obtained along the stretching coordinate for ALA_10_ in implicit solvent (see, for example, Ref. [38]), there are two significant advances in the present study: (i) We have obtained the PMF for the vacuum, implicit solvent and explicit solvent cases using a consistent force field for the peptide thereby allowing for a direct comparison of the effects of the varying solvent conditions on the PMF; and (ii) We have obtained the hydrogen-bonding structure along the pathway in each of the three solvent conditions. Below, we compare the the free energy functions of unfolding in explicit solvent simulations with the implicit solvent simulations and find qualitative agreement. Near the native state, the free energy profile of the explicit model is slightly more structured. The most significant effect on the PMF is a lowering of the free energy at the unfolded state of circa 2 kcal/mol in the explicit solvent. An additional major finding is that the hydrogen-bonding profiles are qualitatively similar along the pathway between the implicit and explicit solvent simulations.

## Materials and Methods

The adaptive steered molecular dynamics (ASMD) method, previously developed [33] and benchmarked for both vacuum [34] and explicit solvent [7] conditions for ALA_10_, has been used to obtain the PMF of ALA_10_ in implicit solvent. A second method, FR-ASMD, previously developed and benchmarked for only ALA_10_ in vacuum [34] has also been used to obtain the PMF of ALA_10_ in implicit and explicit solvent. Both approaches are stage-wise implementations of steered molecular dynamics (SMD) as is summarized below. It is notable that other staging algorithms, such as STePS developed by Guttenberg, Dinner, and Weare [39] and the Parallel-Pulling Protocol (PPP) developed by Ngo, [40] have been developed with similar goals in mind: to speed the convergence in obtaining averages and to sample rare events efficiently. The ASMD approaches are preferred here because they provide the PMF (and other observables) directly along a chosen reduced-dimensional path.

### Naive ASMD: Theory and Implementation

In the ASMD method, the overall reaction coordinate is divided into segments and the PMF is calculated over each segement within an SMD-like stage using the Jarzynski equality (JE). [20–22, 41] The reaction coordinate [24] is arbitrarily defined as the z-axis, along which the peptide is oriented from end-to-end and stretched in one direction. After the PMF is calculated along the first segment, the final value in the work that most closely matches the final value in the PMF in that stage determines which coordinates and velocities are selected for all trajectories in the subsequent stage. A new ensemble of distinct trajectories is generated in the latter because each trajectory is pulled in the presence of a Langevin bath with different random number sequences. The use of the so-called JE-criterion to contract the sampling space between stages results in faster convergence of the PMF and reduces the overall number of trajectories that must be calculated in comparison with SMD results. [33]

The work performed on the peptide during these pulling simulations, when performed infinitely slowly, is equal to the Helmholtz free energy difference. With Jarzynski averaging, the velocity of pulling can be increased two to five orders of magnitude (to sample speeds as fast as 100, 10, and 1 Å/ns, for example), while still permitting one to obtain equilibrium free energy differences from finite-time measurements. The benefit of using ASMD is consequently increased sampling efficiency in the sense that fewer trajectories and reduced computational resources are employed to obtain a more highly converged PMF than comparable SMD sampling.

### FR-ASMD: Theory and Implementation

FR-ASMD [34] invokes different choices for the segmentation of the reaction coordinate and the contraction in between each stage. It is similar to naive ASMD in that it requires one to break up the reaction coordinate into segments. During the corresponding stages, the peptide is pulled from some initial distance to a final distance. However, between pulling stages, a constrained equilibration stage is used to construct the initial configuration for the next pulling stage. During the constrained-relaxation stage, the end points of the peptide are fixed to their initial positions. As no work is done on the peptide during the constrained relaxation, the Jarzynski average is unaffected by this relaxation. In relaxing the structures with large energy deviations from the minimum energy structures, however, strains that could have skewed the subsequent distributions are removed. This contraction of the initial sample space for a given pulling stage has a similar effect as the more extreme contraction (to a single structure) implemented in naive ASMD, but without incurring any error in the free energy average. It comes at the price, however, that one must integrate the system for a time longer than the relaxation times of the slowest mode orthogonal to the fixed pulling coordinate.

### Small Peptide Model

In this work, we are primarily interested in how well our methods perform in implicit solvent and how those results compare to our benchmarked work in explicit solvent. The small peptide, ALA_10_, satisfies the requirements of the implicit solvent model for our study because it is uncharged and hydrophobic. It also has interactions with explicit solvent water molecules, which we have studied previously [7] and recalculated here at a higher level of detail. The intrapeptide and peptide-solvent hydrogen bonds can be used as a tool for comparison between the explicit and implicit solvent models. This is a significant test of the implicit model, which does not explicitly include the specific locations and response of hydrogens in the solvent.

As remarked above, our methods are focused on the benchmark ALA_10_ peptide because it offers a challenge to the solvent models with respect to the determination of the energetics and the structure. (Throughout this work, we use ALA_10_ to refer to the linear peptide containing ten alanines with the acetylated N-terminus and amidated C-terminus endcaps as in Ref. [18] and which contains only 104 atoms.) ALA_10_ lends itself to numerical simulation with low cost. It is notable that there continues to be a debate in the literature over the solubility of these peptides. [42–44] Short-time numerical equilibration confirms that ALA_10_ in vacuum [18] and in explicit solvent relax to a helical form using the force fields in this work, and provides the starting structures for all of the pulling simulations. Counter arguments numeically observed stability of the folded structure in solution arise from observations that short alanine homopolymers can aggregate into *β*-sheets [45, 46] and that a short sequence of alanine in silk protein can form *β*-sheet aggregates. [47, 48] A possible explanation to these contradicting observations is that the native state of ALA_10_ depends heavily on the environment of the peptide. Regardless, our methods do not entirely presume or rely on the stability of the *α*-helix as all calculations are performed under the constraint of an isolated peptide to a particular extension while submerged in water.

### Simulation Protocols in Vacuum and Explicit Solvent

**Fig 2:**
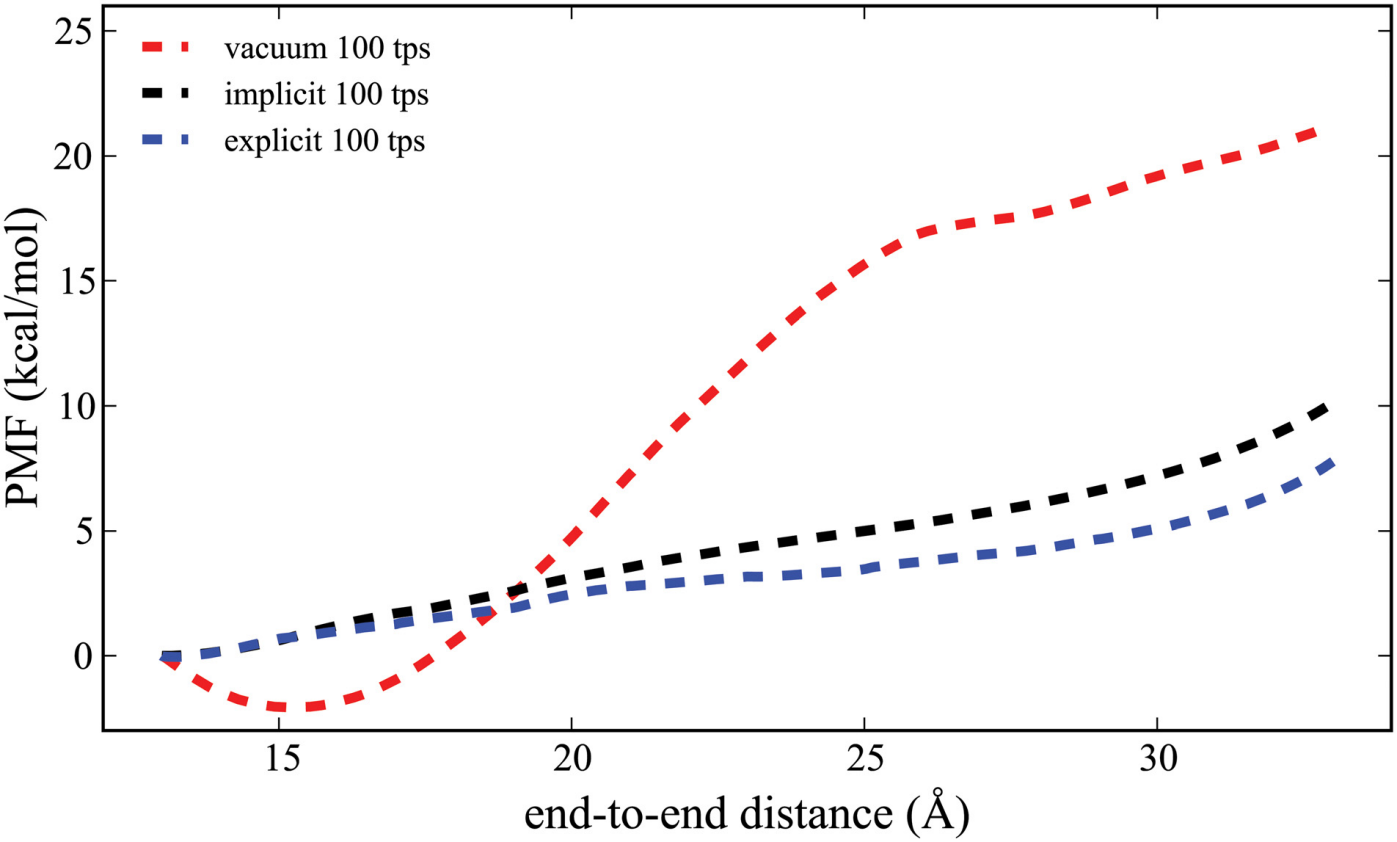
Comparison of the PMF for ALA_10_ in implicit solvent (black curve) to the vacuum (red curve) and explicit solvent (blue curve) results. These PMFs are generated using ASMD at a pulling speed of 1 Å/ns.

**Fig 3:**
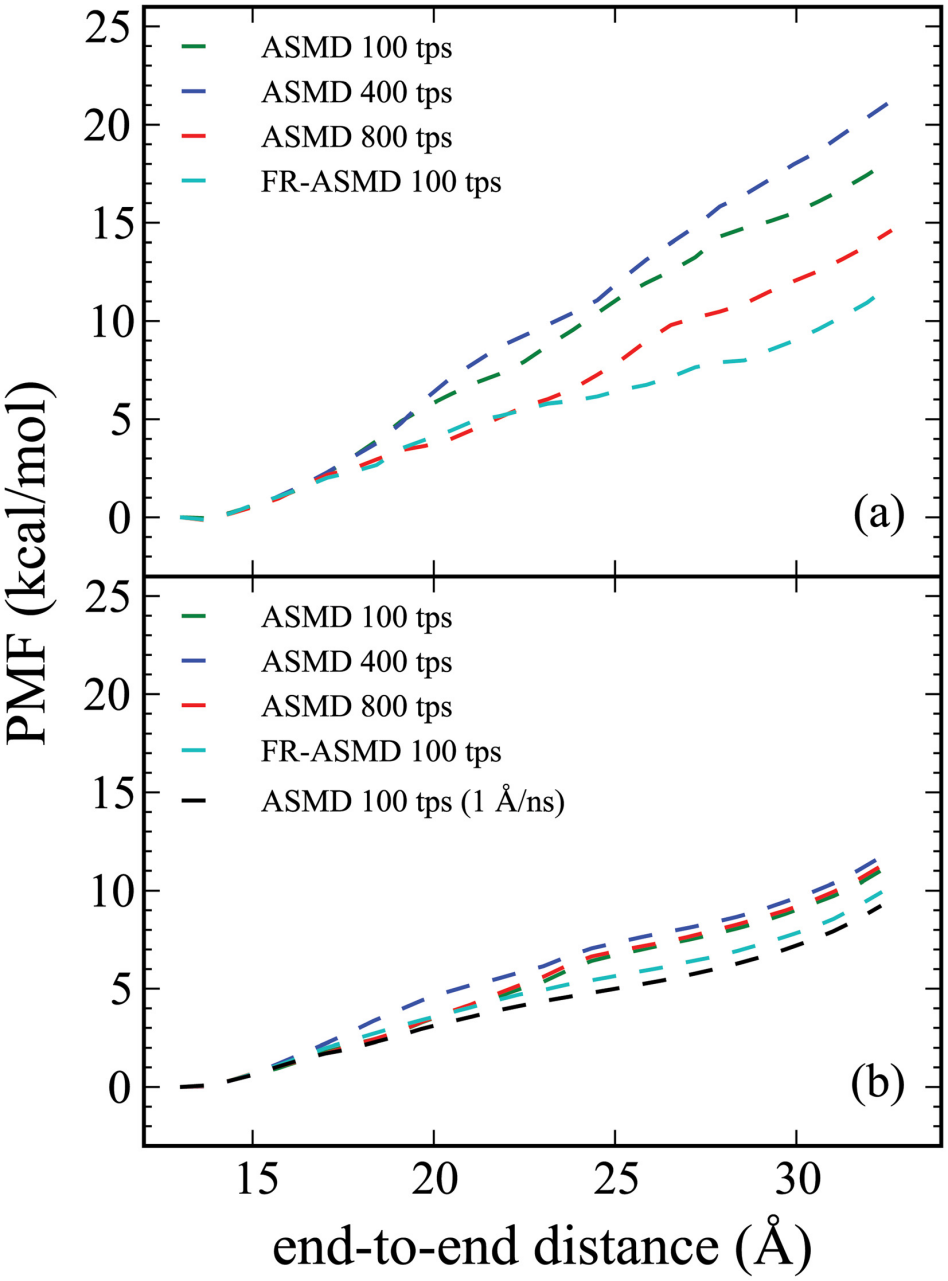
Convergence of the PMF along the ALA_10_ stretching mode in the presence of an explicit solvent calculated using both the ASMD and FR-ASMD methods. The top panel (a) includes the PMFs obtained at 100 Å/ns pulling velocity. The bottom panel (b) includes the PMFs obtained using ASMD and FR-ASMD at 10 Å/ns pulling velocity. In both panels, the red, royal blue, and green curves correspond to ASMD calculations using 800, 400, and 100 trajectories per stage (tps), respectively. A slower pulling ASMD PMF obtained at 1 Å/ns but with only 100 tps is shown in purple in the bottom panel. An even slower SMD simulation obtained a 0.1 Å/ns but with still fewer trajectories (10 tps) is shown in yellow in the bottom panel. In both panels, the light blue curve is the result of the FR-ASMD for 100 tps using 100 ps relaxation stages at the fastest velocity of the ASMD simulations also shown in the corresponding panel.

**Fig 4:**
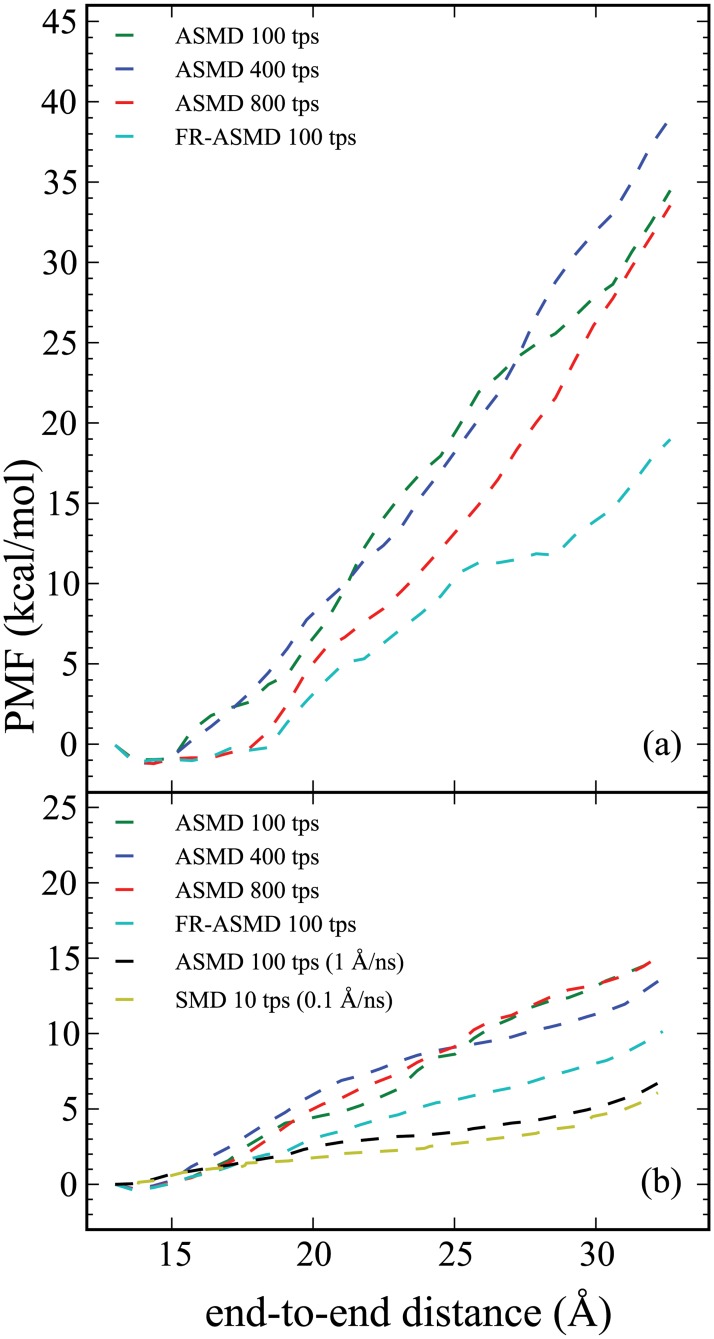
Convergence of the PMF along the ALA_10_ stretching mode in the presence of an implicit solvent calculated using both the ASMD and FR-ASMD methods. All graphs are labeled as in Fig 3.

The ASMD calculations of the PMF are all accomplished using in-house scripts overlaid on the NAMD [49] integrator. All equilibrations and simulations reported here use the CHARMM22 [50] force field at a temperature of 300 K. All calculations are performed under *NVT* conditions. For each PMF (such as those shown in Figs 2, 3 and 4), the reaction coordinate is broken into 10 equal segments, each with 2 Å in length. The overall reaction coordinate corresponds to a total of 20 Å pulling distance, from the folded structure at 13 Å to the fully extended structure at 33 Å. The distance is defined as that between the C_*α*_ of the 1st and 10th residues. The pulling force constant, *k*, is set to 7.2 kcal/mol which is consistent with previous work. [7, 18, 19, 34]

The energetics of ALA_10_ in vacuum and explicit solvent obtained previously using ASMD, [7, 34] have been reproduced both to benchmark the more general scripts used here and to serve as a comparison to the results found in implicit solvent. Equilibration of ALA_10_ for 1 ns in vacuum gives rise to a bound helical structure which is similar to that found by Schulten and coworkers [18] despite the present use of a more recent force field. The end-to-end distance, measured between the carboxyl nitrogens associated with the two terminal ends, is roughly 13.4 Å. In the propagation of ALA_10_ using NAMD, the cutoff, switch, and pair list distances are taken to be 12 Å, 10 Å, and 13.5 Å respectively. In all cases, the time step is 2 fs, and temperature is maintained using a Langevin damping coefficient of 5 ps^−1^.

In explicit solvent, ALA_10_ is solvated in a 20 Å × 20 Å × 50Å rectangular cuboid with the long-axis arbitrarily labeled as *z*. The initial positions of ALA_10_ are taken from the vacuum structure with the nitrogen atom of the N-terminus and the nitrogen atom of the cap of the C-terminus placed on the *z* axis. The box is filled with 897 TIP3P [51] water molecules (as implemented in the psfgen routine associated with NAMD [49]) for a total simulation size of 2795 atoms in accordance with the density of water under these conditions. While this is a reduction of the solvent box from our previous simulation study, we have reproduced the previous data using this reduced system and have saved significant computational costs. We have ensured the equilibration of our system through use of Root Mean Square Deviation analysis and through evaluating density, energy, volume, and pressure fluctuations. In previous work, [7] the nitrogen atoms at each end were held fixed while the rest of the protein and solvent were allowed to relax. The resulting initial structure was then used for all the ASMD pulling simulations allowing for direct comparison with the vacuum results but at the possible sacrifice of complete relaxation of the initial structure. In the present work, all atoms (peptide and water molecules) are initially equilibrated for 1 ns so as to ensure that the initial peptide structure is near a local minimum in parallel with a similar protocol for the implicit solvent simulations. This relaxation procedure results in a slightly different (but still *α*-helical) structure with an end-to-end-distance of 12.72 Å, which is slightly smaller than the vacuum structure. Thereafter, the system is reoriented so that the nitrogen atoms at the ends of the peptide lie on the z-axis. This ensures that the pulling of the peptide occurs parallel to the long axis of the water box.

For the FR-ASMD simulations, 100 ps constrained relaxation stages are used between each pulling stage. Each trajectory is propagated (separately but in parallel) with the end-to-end distance (associated with the nitrogen positions) constrained to their respective initial values. This ensures that no external work is being performed along this (or any) coordinate. In previous work, [34] constrained relaxation stages of greater than 50 ps were demonstrated to be sufficient to obtain nearly converged (and correct) PMFs in the case of ALA_10_ in vacuum. Therefore, the choice of equilibration time (100 ps) was made without additional convergence checks. It is possible that a longer relaxation time would be necessary in the presence of solvent, and this comprises one possible source of approximation in the FR-ASMD results obtained below.

### Simulation Protocols in Implicit Solvent

For the implicit solvent calculations, the equilibrated vacuum structure was solvated using Generalized Born Implicit Solvent (GBIS) as implemented in NAMD. The cutoff, switch, and pair list distances are also set to 18, 16, and 20, respectively. All propagations are performed using a 2 fs time step and a Langevin damping coefficient of 5 ps^−1^. The peptide is initially equilibrated before the beginning of the first stage of simulations for 1 ns. As in the explicit solvent, the constrained relaxation stages in the FR-ASMD simulations are propagated for 100 ps.

### Hydrogen-Bonding Profiles

Hydrogen-bonding profiles have also been obtained for vacuum, implicit, and explicit solvent. The intrapeptide hydrogen bonds in ALA_10_ can be readily obtained for the three cases: vacuum, implicit solvent, and explicit solvent. The intrapeptide hydrogen-bonding is evaluated using the python library MDAnalysis. [52] Hydrogen bonds are designated when the donor and acceptor atoms are within 4 Å of each other and the angle formed between the hydrogen, donor, and acceptor is greater than 140°. The average hydrogen bond count is weighted according to the work averages from the simulations, consistent with previous studies. [7, 34] The peptide-solvent hydrogen bonds can be obtained readily in the explicit solvent case. However, there is no solvent in the vacuum case and the specific positions of the water molecules in the implicit solvent case are not represented. In order to capture the effective hydrogen-bonding to solvent, we take an ensemble of peptide structures along the pathway, individually hold each fixed, and equilibrate water molecules at the appropriate density around it for 100 ps under NVT conditions. The peptide-peptide hydrogen-bonds for each such structure remains the same (because the peptide structure is unchanged), but an effective number of peptide-solvent hydrogen bonds can now be obtained following the same procedure as performed in the all-atom case.

## Results

The energetics of the directed unfolding of ALA_10_ has been explored using two different variants of SMD: ASMD and FR-ASMD. The simulations are performed at 3 different pulling velocities, 100 Å/ns, 10 Å/ns, and 1 Å/ns, and in three different environments, vacuum, implicit, and explicit solvent. For each of these pulling velocities and environments, the number of trajectories was varied from 100 to 800 trajectories per stage so as to achieve convergence in the energetics.

**Fig 5:**
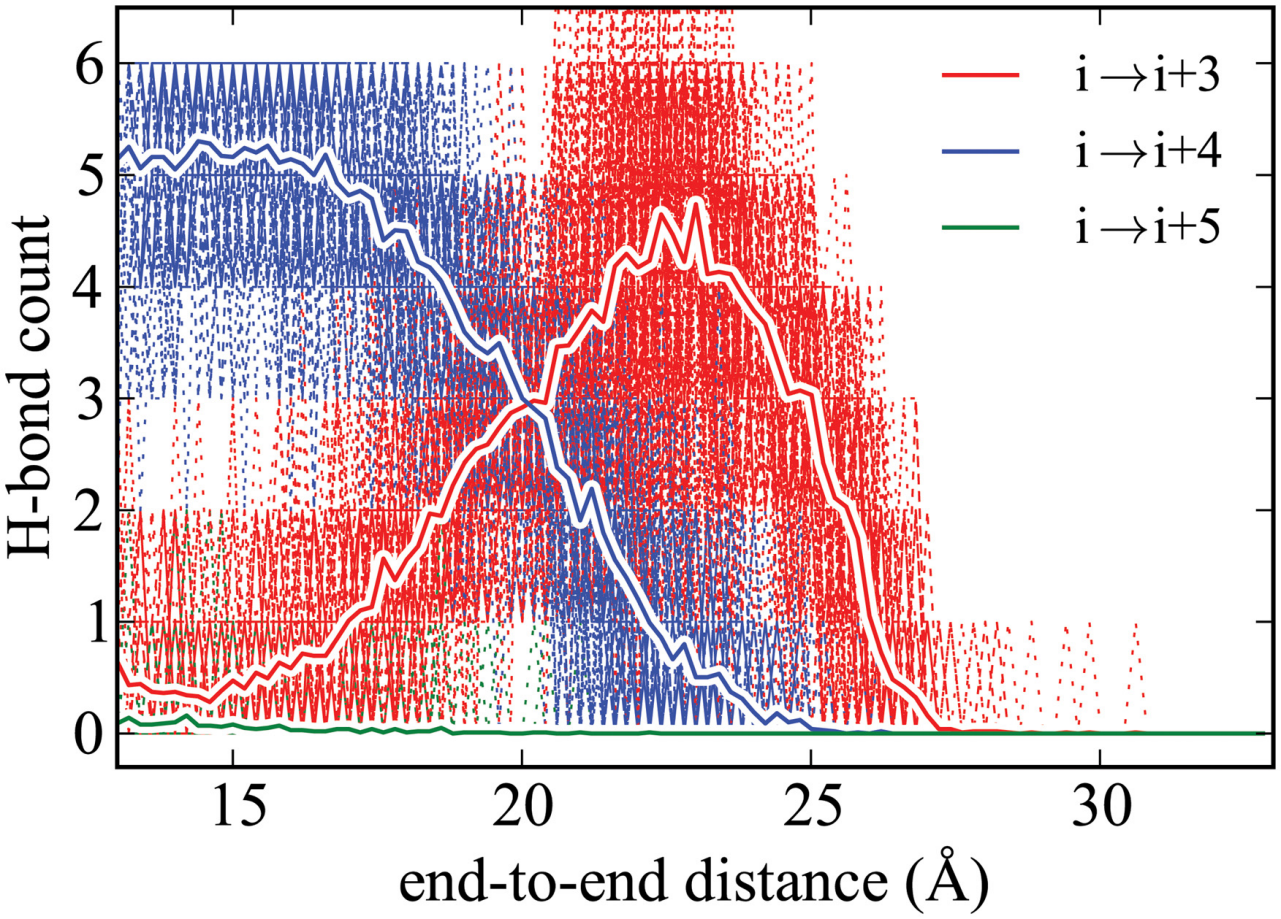
Hydrogen-bonding profile for ALA_10_ in vacuum calculated using ASMD at simulation conditions, 1 Å/ns with 100 tps, differing with those of our earlier work. [34] The red, blue and green solid lines corresponds to the average number of *i* → *i* + 3 (3_10_-helix), *i* → *i* + 4 (*α*-helix), and *i* → *i* + 5 (*α*-helix) contacts, respectively. They are overlaid onto scatter plots showing the range of values visited by the specific trajectories, thereby indicating the error in the averages.

**Fig 6:**
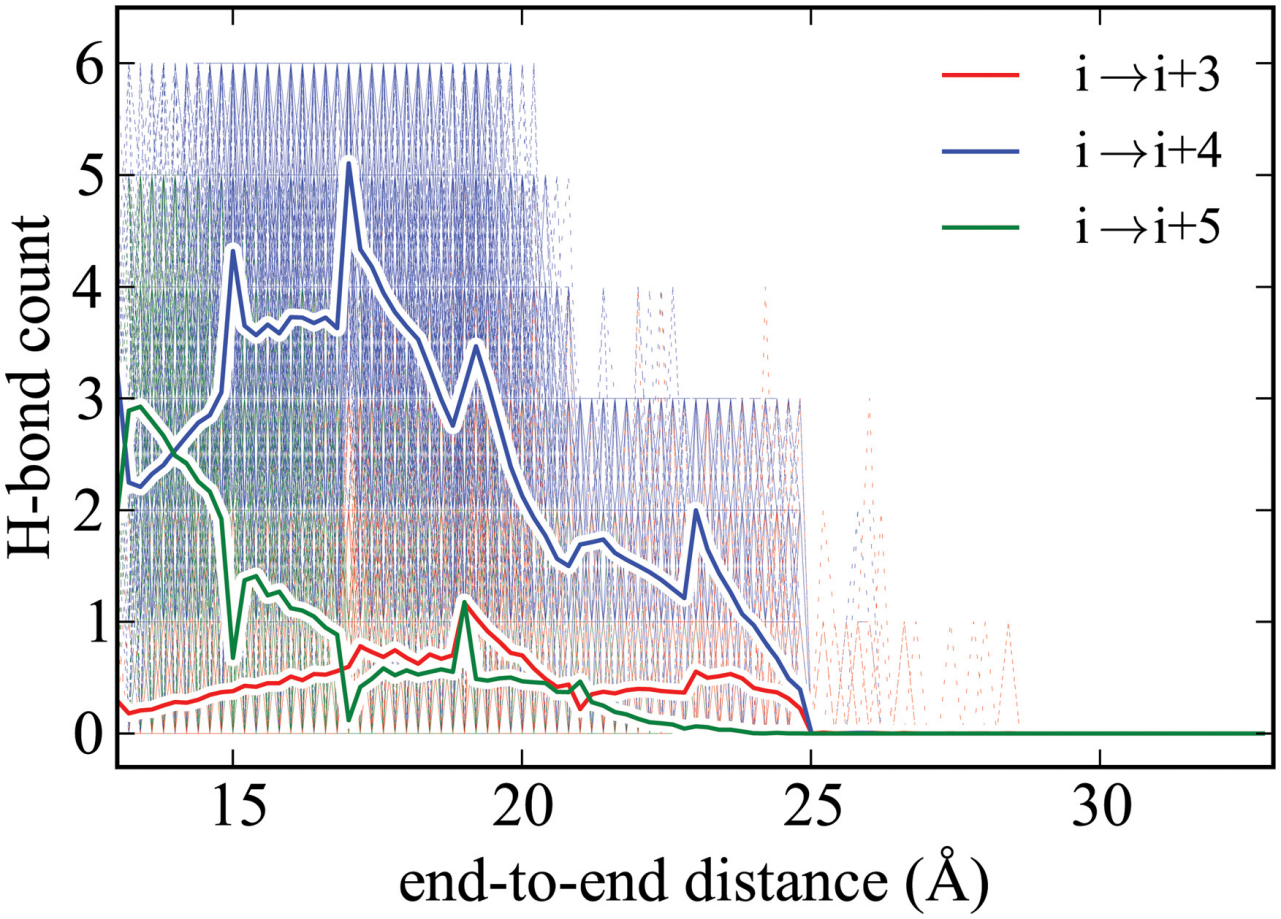
Hydrogen bond profile of ALA_10_ in implicit solvent using ASMD at 10 Å/ns with 800 tps. All curves are shown as in Fig 5.

**Fig 7:**
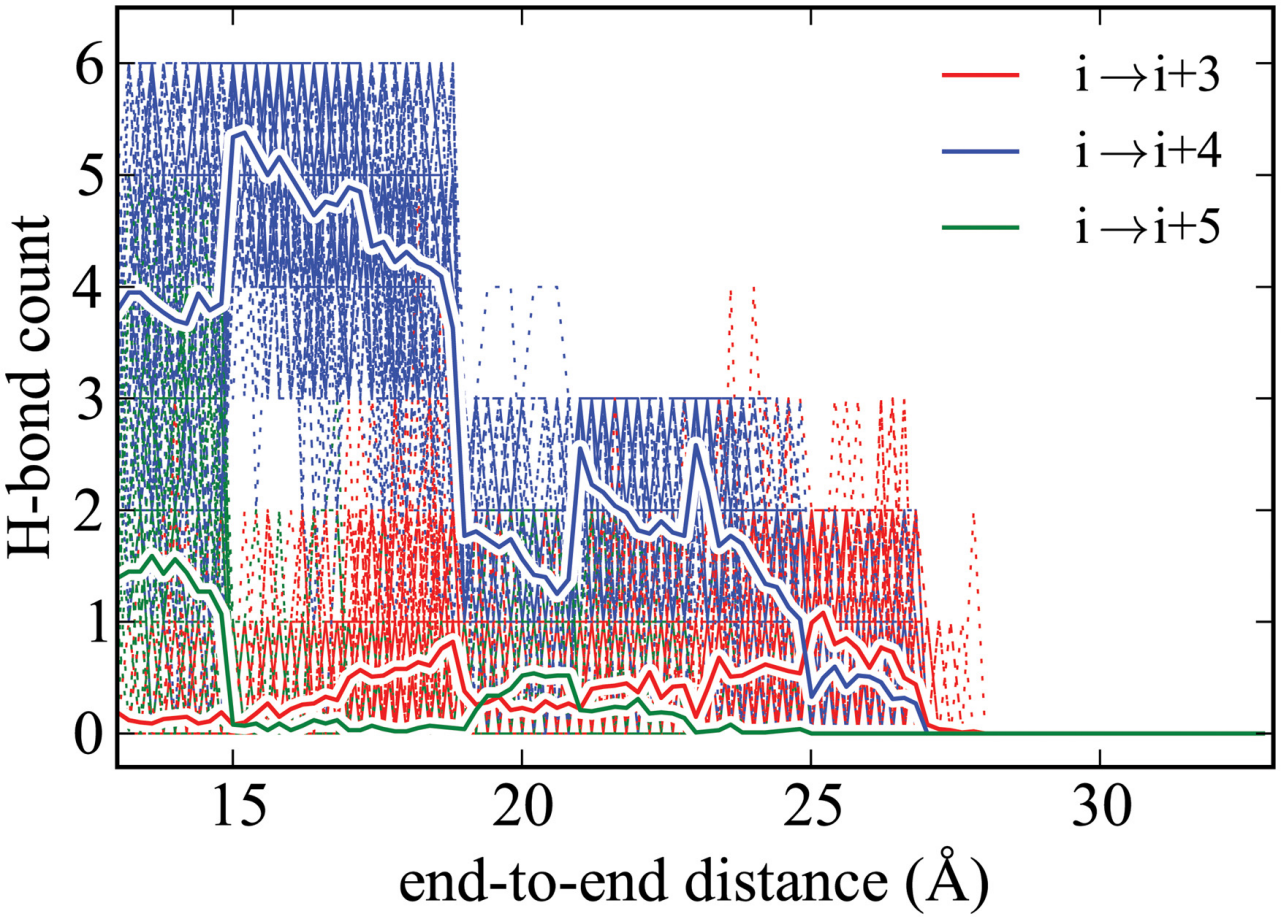
Hydrogen-bonding profile for ALA_10_ using ASMD at 10 Å/ns with 100 tps recalculated here for the explicit solvent [7] case. All curves are shown as in Figs 5 and 6.

**Fig 8:**
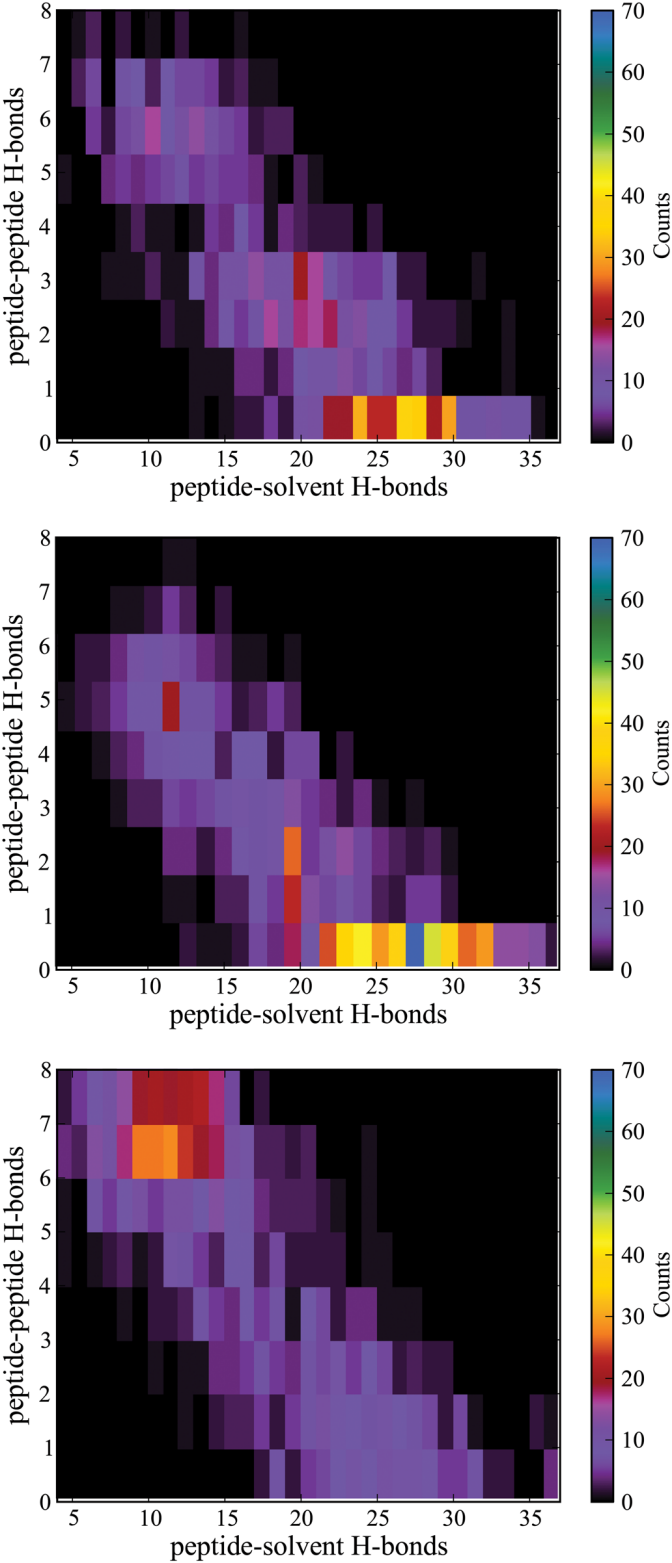
Two-dimensional histogram of peptide-peptide and peptide-solvent hydrogen bonds for ALA_10_ structures in explicit, implicit, and vacuum along an ASMD pulling path. The color scale on the right from black to blue indicates the density of the points for that region of the map.

The results presented here include the determination of the PMF using naive ASMD and FR-ASMD at varying pullng speeds in varying solvent conditions. Specifically, Fig 2 provides the direct comparison between the converged PMF for ALA_10_ in vacuum, implicit solvent, and explicit solvent. We used a pulling velocity of 1 Å/ns velocity for the vacuum and explicit solvent simulations, which is 10 times slower than our previous work, so as to confirm convergence with respect to the pulling velocity. [34] The convergence of the potentials are illustrated in Figs 3 and 4, for the explicit and implict solvents, respectively. The hydrogen-bond profiles along the ASMD pull are shown in Figs 5, 6 and 7, for ALA_10_ in vacuum, implicit solvent, and explicit solvent. A histogram of the intrapeptide hydrogen bonds as correlated with the actual or effective peptide-solvent hydrogen bonds in each of these three cases is shown in Fig 8. In the vacuum and implicit solvent cases, the peptide-solvent hydrogen bonds are *effective* in the sense that the relaxation of an all-atom water solvent around the fixed peptide is used to infer the hydrogen bonds to a solvent following the procedure described in Materials and Methods.

## Discussion

### Comparison of the energetics of ALA_10_

A central result of this paper is summarized in Fig 2: the PMF for ALA_10_ in the presence of an implicit solvent is seen to much more closely resemble that of the explicit solvent than the vacuum. Therein, the PMF for the explicit case is considerably lower at the longest extension of the peptide (33 Å), by approximately 12 kcal/mol, than the vacuum case, as was previously reported. [7] Although not shown, the convergence and PMFs studied in the vacuum case were seen to be in near perfect agreement with that reported in earlier work at a velocity of 10 Å/ns. [34] The Δ*G* from native to unraveled protein in an explicit solvent, as shown in Fig 3, is 7 kcal/mol which is lower than that found at the faster pulling speeds but the differences have narrowed toward convergence. These results can also be compared to the most converged PMF reported by Tomberli and coworkers [38] using drift-oscillatory steering which found a small lowering in the free energy difference relative to the vaccum result at just below 20 kcal/mol. This suggest that the nonequilibrium ASMD simulations are able to sample pathways with significantly lower energy penalties to the stretching of ALA_10_.

Recently, Gumbart and coworkers [8] found a different form for the PMF of ALA_10_ in solvent using adaptive biasing forces and umbrella sampling with the weighted histogram analysis method using the CHARMM27 and CHARMM36 force fields. In their one-dimensional vacuum PMFs, they found an overall change in the PMF from folded to unfolded in solution to be on the order of 20 kcal/mol which is comparable to what we and others have found for the same process in vacuum. We find here that the addition of solvent, whether implict or explicit, gives rise to significant stabilization of the pathway bringing the energy change down to circa 5 kcal/mol as would be expected by the presence of stabilizing hydrogen bonds in the open structure. Gumbart and coworkers also obtained 2-dimensional potentials of mean force for the folding of ALA_10_ in explicit solvent. They define their reaction coordinates as the end-to-end distance of the peptide (similar to the work we describe here) and as the degree of *α*-helical content of the peptide. An effective one-dimensional PMF with respect to end-to-end length resulted from the summation over the discrete orthogonal variable. There are significant differences between the 1D-PMF obtained by Gumbart and coworkers and the ones we report in this work. They report a second minimum in the unfolded conformation of the peptide. The differences are likely resulting from (i) sampling, (ii) the choice of reaction coordinate, and (iii) the imposed constraints. In particular, the coordinate orthogonal to the reaction coordinate imposed by Gumbart and coworkers is discrete, but global, and may thereby include structures that are very far (conformationally) to the on-pathway (with respect to the stretching) structures that we are describing. Our nonequilibrium trajectory sampling procedure necessarily suppresses structures that are very far from the pathway which would presumably not be strong contributors in the course of a pulling experiment. These effects likely account for the differences in the free energies that we find along the pathway in comparison with those found in the 2D PMFs by Gumbart and coworkers.

For both implicit and explicit solvent, the decrease of the pulling velocity from 100 to 1 Å/ns leads to convergence of the PMF due to the ability of the peptide to explore more conformational space given more simulation time. The much slower SMD simulation in explicit solvent (0.1 Å/ns) also shown in Fig 3 is obtained from only 10 trajectories. While this is likely not enough to obtain full convergence, it does provide a rough benchmark for a possible lower bound to the energetics. (It is notable that the PMF for ALA_10_ in vacuum performed at this velocity was converged with only 1 trajectory using SMD. [19]) Nevertheless, in explicit solvent there is near agreement between the ASMD (at 1 Å/ns) and the SMD (at 0.1 Å/ns) suggesting that the former can also serve as a near benchmark for the ASMD simulations. The convergence of the ASMD PMFs in comparison to the near benchmark FR-ASMD result is shown in Fig 4. The PMF at 10 Å/ns in implicit solvent converges to that at 1 Å/ns more rapidly than in explicit solvent. Thus the lack of explicit solvent fluctuation and relaxation modes in the implicit solvent leads to less spread in the sample space of the nonequilibrium trajectories.

The use of FR-ASMD significantly lowers the PMF achieved by ASMD in both the implicit and explicit solvent cases. FR-ASMD seems to do particularly well at the faster velocity 100 Å/ns in the sense of leading to the most dramatic stabilization in the PMF. (Refer to the light blue curves in Figs 3 and 4 for the FR-ASMD results.) There are several reasons why the FR-ASMD method decreases the PMF so dramatically. Perhaps the most significant of which is the amount of simulation time that has been added back to the simulation. In the case of the 100 Å/ns pulling simulations, the equilibration time amount to approximately 1 ns, which is comparable to the amount of time required for a corresponding SMD or ASMD simulation obtained at a pulling speed that is ten times slower. It is notable that this discrepancy is not as severe at the slower pulling speeds, when the equilibration time becomes a smaller relative component, but there the gain in accuracy is not as dramatic. Secondly, the contraction to only a single structure from each subsequent stage in ASMD may, in general, also be too stringent for the collapse of the work distributions. Inclusion of the relaxation stages with no contraction to a single structure allows the peptide time to reorient itself with the surrounding solvent molecules, mimicking a slower pulling velocity.

A comparison of the relative computational costs of the ASMD methods is also instructive. All propagations in this work were performed on NAMD in single-core mode on 2.0 GHz AMD Opteron 6128 processors, and reported timings are relative to this resource. The typical CPU time required to propagate a single trajectory (per stage) in explicit solvent at 100 Å/ns and 10 Å/ns pulling is 9 minutes and 93 minutes, respectively. For implicit solvent, the computational time is dramatically reduced to 1 minute and 10 minutes, respectively. FR-ASMD simulations have the same time requirements as in the above cases for the pulling stages, but require 9 additional constrained relaxation stages (in the present case of 10 pulling stages as used throughout the simulations reported here.) In explicit and implicit solvent, each such constrained relaxations stage required an additional 182 and 24 minutes respectively. This is a substantial penalty, and suggests a need for a more efficient approach for the contraction in ASMD. In summary, naive ASMD is the most efficient of these, but it requires a check on convergence which may limit its efficiency.

### Hydrogen-bonding profiles

The hydrogen-bonding profiles, in conjunction with the PMFs, reveal interesting structural characteristics of the unfolding of ALA_10_. As previously reported, [34] the change in the PMF between the end points for ALA_10_ in vacuum is higher than that found in solvent partly because of differences in the hydrogen-bonding structure. During the unfolding of ALA_10_ in vacuum, the number of *i* → *i* + 4 (*α*-helix) contacts, where *i* refers to the index of the residue, decreases steadily as shown in Fig 5 after initially being insensitive to the extension. The loss of *i* → *i* + 4 contacts appears to be coupled with the onset of *i* → *i* + 3 (3_10_-helix) contacts just before an end-to-end distance of 18 Å. The number of *i* → *i* + 3 contacts reache a maximum at an extension of 23 Å, and are thereafter precipitously lost. There is no formation of *i* → *i* + 5 (*π*-helix) contacts. The exchange of *i* → *i* + 4 contacts with *i* → *i* + 3 contacts was seen earlier to lead a near constant total number of hydrogen bonds for the first 15 Å in extension between the ends of the peptide.

This behavior is in stark contrast with the hydrogen-bonding profile observed in explicit solvent shown in Fig 7. In explicit solvent, there is no formation of the *i* → *i* + 3 contacts while the *i* → *i* + 4 contacts are lost. [7] This is due to the presence of water molecules that are able to hydrogen bond with the peptide as it is stretched. Unlike in the vacuum case, there is slight formation of the *i* → *i* + 5 (*π*-helix) contacts. This finding is corroborated by a similar conclusion about the role of hydrogen-bonding in Human Amylin seen earlier by Skinner and coworkers. [3] Namely, that hydrogen bonds from the solvent significantly contribute to the stabilization of the unfolded helix in random coil.

As shown in Fig 6, we find only small differences in the hydrogen-bonding trend between the implicit and explicit solvent cases in accord with the good agreement seen earlier between their PMFs. In both cases, the trend is also unlike that seen in the vacuum, but this is to be expected as the solvent provides sufficient solvation to limit the dramatic formation of *i* → *i* + 3 contacts seen in the vacuum case. The main difference, albeit small, between the implicit and explicit solvent hydrogen-bonding profiles appears to be due to the fact that the initial equilibrium structures are quite different. Namely, ALA_10_ in implicit solvent appears to have significant *π*-helical structure as observed from the presence of a larger number of *i* → *i* + 5 contacts comparable to the number of *i* → *i* + 4 contacts. The loss of *π*-helical structure with increased end-to-end distance initially results in *α*-helical structure rather than no structure at all.

The role of hydrogen bonds between the peptide and water can be monitored readily in an explicit solvent by enumerating the contacts along the pathway. Fig 8(top) displays a histogram of the peptide-peptide and peptide-solvent hydrogen bonds (for one thousand structures) along the most favored pathway (as determined by the JE-criterion) as the peptide is stretched. Unfortunately, such a procedure is not readily available in an implicit solvent (and certainly not in vacuum), making it difficult to confirm the hypothesis that the hydrogen-bonding to solvent is somehow being characterized appropriately by the implicit solvent as suggested by the analysis above. Instead, we follow the procedure outlined in the Materials and Methods to obtain the effective number of peptide-solvent hydrogen bonds that would be created if the solvent were to be equilibrated around the fixed peptide structures. The histograms for the peptide-peptide and effective peptide-solvent hydrogen bonds along the most favored pathway (as determined by the JE-criterion) as the peptide is stretched in the implicit solvent and vacuum are shown in Fig 8(middle) and 8(bottom), respectively.

The similarity between the histograms for explicit (top) and implicit solvent (middle) in Fig 8 is startling. In all three cases, the number of intrapeptide hydrogen bonds decreases steadily as peptide-solvent hydrogen bonds increase. However, the relative abundance is strongly shifted toward the case when many peptide-solvent hydrogen bonds are favored only in the cases of the implicit and explicit solvation. In the vacuum, the largest abundance of hydrogen bonds occurs in the regime when they are primarily intramolecular. That is, the opening of the peptide is not followed by structures that would be readily solvated through hydrogen-bond contacts. This should not be surprising for the vacuum case because the structures are formed in absence of water. However, the fact that the implicit solvent induces structures that readily admit hydrogen bonds in near proportion as those seen in the explicit solvent case further suggests that the former does indeed include hydrogen-bonding (implicitly) at a level of detail that might not have been anticipated.

Thus, the overall pathway seen in the implicit solvent appears to involve the loss of internal helical structure that is predominantly replaced by solvent contacts in agreement with that observed in the case of explicit solvent. This method of solvating and equilibrating the implicit and vacuum structures is simply used as a tool to understand how the structures respond to solvation.

## Conclusion

This work provides a comparison of the potentials of mean force and hydrogen-bonding profiles of the forced unfolding of ALA_10_ using primarily two different methods, ASMD and FR-ASMD, at different pulling velocities and in three different environments. Using a variety of velocities and environments, albeit on a simple single-motif peptide, benchmarks for energetics and structure in small molecule pulling simulations have been obtained.

Perhaps surprisingly, there is qualitative agreement between the potentials of mean force and the overall hydrogen-bonding pathway for ALA_10_ stretching in implicit and explicit solvent. However, the equilibrium structure of ALA_10_ in implicit and explicit solvent differ only slightly with the implicit solvent giving rise to a mix of *π*-helical and *α*-helical structure while the explicit solvent gives rise to primarily *α*-helical structure. This suggests that the implicit solvent model slightly over-emphasizes contact between the solvent and peptide. Meanwhile, the fact that the pathways in the presence of an implicit or explicit solvent differs dramatically from that in vacuum illustrates the importance of the use of solvent models (either explicit or implicit) so as to capture the actual dynamics of the stretching. A detailed view of the effective peptide-water hydrogen-bonding (by way of solvating explicit water to the fixed protein structures) in an implicit solvent has also shown strong agreement to the actual peptide-water hydrogen-bonding in an explicit solvent. This observation provides further evidence that hydrogen-bonding is effectively encoded in the implicit model. Thus, the implicit models are capturing the stabilization of the structure with respect to hydrogen bonds in a manner that is analogous to the explicit water. As such, the PMFs obtained from the implicit and explicit water models were qualitatively and quantitatively similar.

Finally, ASMD has been seen to produce a converged PMF of ALA_10_ with fewer trajectories at comparably faster velocities than traditional SMD, and does best in the vacuum case. The use of FR-ASMD, which includes intermediate stages for relaxation without using any type of trajectory selection, does improve the PMF substantially in comparison with naive ASMD at equivalent pulling velocities in the implicit and explicit solvent cases. This suggests a need for improved trajectory selection schemes in ASMD to address the increased complexity of the ensemble space in the presence of solvent. Improved contraction schemes that avoid the expense of the propagation of trajectories while not being limited to the limitations of a single representative of the nonequilibrium ensemble are therefore of strong interest. [53]
